# Treatment of alcohol use disorder in alcohol-associated liver disease: A meta-analysis

**DOI:** 10.1097/HC9.0000000000000686

**Published:** 2025-04-30

**Authors:** Ashwani K. Singal, Wanyu Zhang, Akshay Shetty, Arpan Patel, Shaikhoon Mohammed, Prabha Bhandari, Mohamed Abdallah, Vatsalya Vatsalya, Lorenzo Leggio, Maiying Kong

**Affiliations:** 1 Division of Gastroenterology Hepatology Nutrition, University of Louisville School of Medicine, Louisville, Kentucky, USA; 2 UofL Health Jewish Hospital, Louisville, Kentucky, USA; 3 Trager Transplant Center, Louisville, Kentucky, USA; 4 Robley Rex VA Medical Center, Louisville, Kentucky, USA; 5 Department of Medicine and Division of Gastroenterology and Hepatology, University of California Los Angeles, Los Angeles, California, USA; 6 Department of Hospital Medicine, Emory University, Atlanta, Georgia, USA; 7 Division of Infectious Disease, University of Louisville School of Medicine, Louisville, Kentucky, USA; 8 Division of Gastroenterology, Cleveland Clinic, Ohio, USA; 9 Department of Medicine, University of Louisville School of Medicine, Louisville, Kentucky, USA; 10 Clinical Psychoneuroendocrinology and Neuropsychopharmacology Section, Translational Addiction Medicine Branch, National Institute on Drug Abuse Intramural Research Program and National Institute on Alcohol Abuse and Alcoholism Division of Intramural Clinical and Biological Research, National Institutes of Health, Bethesda, Maryland, USA; 11 Department of Behavioral and Social Sciences, Center for Alcohol and Addiction Studies, Brown University, Providence, Rhode Island, USA; 12 Department of Medicine, Division of Addiction Medicine, School of Medicine, Johns Hopkins University, Baltimore, Maryland, USA; 13 Department of Neuroscience, Georgetown University Medical Center, Washington, District of Columbia, USA; 14 Department of Bioinformatics and Biostatistics, School of Public Health and Information Sciences, University of Louisville, Kentucky, USA; 15 Brown Cancer Center, University of Louisville, Louisville, Kentucky, USA

**Keywords:** ALD, AUD, cirrhosis, MAUD, relapse, transplant

## Abstract

**Background:**

To examine alcohol use disorder (AUD) treatment in patients with alcohol-associated liver disease (ALD) on alcohol relapse and liver-related outcomes.

**Methods and Results:**

Twenty-five eligible studies on 93,899 (33,834 AUD intervention) patients with ALD were analyzed. Data presented as HR, with a 95% CI. Of 14 studies in patients with ALD outside the liver transplantation (LT) setting, pooled data from 7 randomized controlled trials (RCTs) showed that AUD treatment reduces alcohol relapse by 73% (HR: 0.27, 95% CI: 0.15–0.46) with any treatment and by 77% (HR: 0.23, 95% CI:0.14–0.39) with medications in 5 RCTs on 322 (186 intervention) patients. AUD treatment from observational studies was associated with reduced readmission (5 studies) by 48% and decompensation (2 studies) by 52%, but not patient mortality (3 studies). Data showed moderate to high heterogeneity, without publication bias. Analysis of 8 observational studies on LT recipients showed that AUD treatment reduced alcohol relapse in the post-LT period by 59%, with 58% and 60% reduction using integrated and nonintegrated models, respectively. AUD treatment among LT recipients was associated with a reduction in patient mortality by 56% in 3 observational studies, but not in 2 RCTs (HR: 0.82, 95% CI: 0.38–1.79). Pooled data were homogeneous in the analysis of alcohol relapse but showed moderate heterogeneity in analyzing patient mortality.

**Conclusions:**

Available data on AUD treatment in patients with ALD improves abstinence and liver-related outcomes both outside and within LT settings. RCTs are needed to examine (a) medications in patients with ALD to examine the benefit of alcohol relapse and patient outcomes and (b) the benefit of integrated multidisciplinary treatment to manage the dual pathology (AUD and liver disease).

## Graphical abstract

**Figure:**
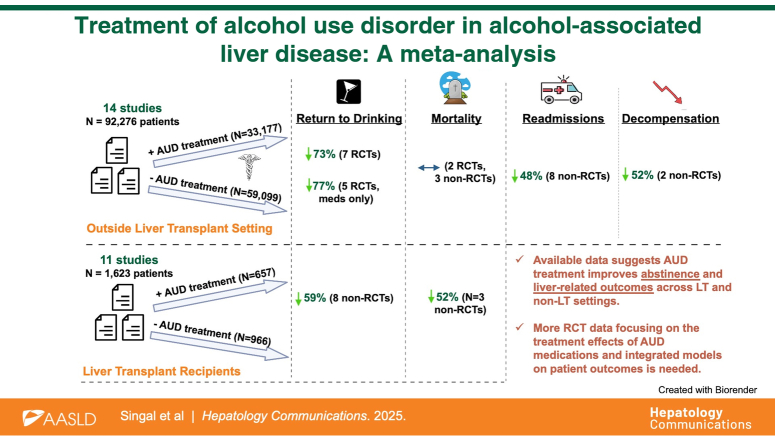

## INTRODUCTION

In the United States, alcohol contributes to 48% of inpatient care and 27.3% of deaths related to cirrhosis, with the direct cost of patient care of $22.7 billion.^1^ A total of 75%–80% of patients with alcohol-associated liver disease (ALD) have moderate to severe alcohol use disorder (AUD), which is negatively associated with personal/social responsibilities and with physical/mental health.^2^ ALD is currently the leading indication for liver transplantation (LT),^3^ including early LT for select patients with severe alcohol-associated hepatitis (AH).^4^

Patients with ALD remain at risk for alcohol use recurrence, and this risk persists among LT recipients,^5^ with a prevalence of 11%–50%.^6^ In a more challenging population of severe AH, only 45% of patients were abstinent at 3 months and 30% at 1 year after hospital discharge.^7^ Rates of recurrence to alcohol use after LT for ALD vary from 8% to 20% after 1 year and up to 40%–50% after 5 years of LT.^6^ Alcohol use recurrence is the single most important determinant of long-term outcomes of patients with AUD and liver disease.^8^ Among recipients of LT, heavy alcohol users versus abstinent ones were more likely to have graft findings of steatohepatitis, advanced fibrosis, or cirrhosis, with graft failure and mortality from recurrent ALD or extrahepatic malignancy.^6^

Treatment of AUD reduces alcohol use, development of ALD, and progression to cirrhosis in those with early-stage ALD,^9^ hepatic decompensation, readmissions, and patient mortality in those with cirrhosis and/or AH.^10^ However, in the real world, AUD treatment rates are very low, with only 1.0%–9.7% of patients with ALD, even in an advanced form of cirrhosis, receiving pharmacotherapy for AUD,^10^^,^^11^ due to several barriers at the patient and clinician level.^12^ Further, administrative and logistic issues limit the implementation of integrated multidisciplinary models, leaving siloed (nonintegrated) addiction and hepatology practices to manage patients with ALD.

Clearly, the literature on AUD treatment in patients with ALD is scarce both within and outside the LT setting. In this systematic review and meta-analysis, we pooled the available literature to examine the benefits of AUD treatment within and outside LT setting on alcohol use, liver-related outcomes, and patient survival.

## EXPERIMENTAL PROCEDURES

### Literature search

A computerized literature search was performed by the librarian using the electronic databases PubMed (Medline), Scopus, and Embase for published articles through July 24, 2024. The detailed search strategy is included in the Supplemental Material, http://links.lww.com/HC9/B950.

### Eligibility criteria

Three investigators (Ashwani K. Singal, Akshay Shetty, and Arpan Patel) independently reviewed the literature to select eligible studies for this analysis. Randomized controlled trials (RCTs) and observational retrospective or prospective studies addressing nonintegrated or integrated management of AUD among adults aged ≥18 years with ALD outside the LT setting, and pre-LT and/or post-LT were included in the analysis. Studies comparing AUD treatment (behavioral or pharmacological) with an untreated arm were included. Management of liver disease by a hepatology and addiction team in a colocated clinic or built within the LT center was defined as integrated care, and if the care was provided in separate clinics, this was defined as a nonintegrated care model. Studies were excluded if: (a) published as abstracts or full papers in a language other than English; (b) study design of case report/series, editorial, and narrative reviews; (c) the study population included coinfected patients with HIV, HBV, or HCV infections; or (d) did not report the outcome of interest. Titles, abstracts, and, if needed, full-text were reviewed for the study selection. Any disagreement between the authors was resolved by reviewing the papers in question together and reaching a consensus. Preferred Reporting Items for Systematic Reviews and Meta-Analyses (PRISMA) guidelines were followed in carrying out this meta-analysis.

### Outcomes

The primary outcome was a relapse to alcohol use. Secondary outcomes were hepatic decompensation (ascites, hepatic encephalopathy, or variceal bleeding), readmission, and patient survival. As the outcomes of alcohol relapse and patient mortality were reported at different time points across studies, the ratio of hazard functions between the AUD treatment and control for these outcomes was assumed to be constant over time, an approach that helps to eliminate heterogeneity caused by varying lengths of follow-up across studies.^13^ Alcohol use and its quantification was measured by self-reported use by the patients by AUD Identification Test or Timeline Follow Back tools and was supplemented by nonspecific markers of alcohol use in 1,^14^ carbohydrate-deficient transferrin in 1,^15^ and measurement of urinary ethyl glucuronide in 4 studies.^16^^–^^19^

### Assessment of risk of bias and study quality

Three independent investigators (Shaikhoon Mohammed, Prabha Bhandari, and Ashwani K. Singal) evaluated the methodological quality and bias risk of each study; any discrepancy was resolved through a joint review of the articles in question. The Cochrane risk-of-bias tool was used to assess the risk of bias in randomized trials, non-randomized comparative, and non-comparative studies.

### Data extraction

Four independent investigators (Ashwani K. Singal, Akshay Shetty, Arpan Patel, and Wanyu Zhang) reviewed the articles that met the eligibility criteria. Any discrepancy was resolved by reviewing the article together and reaching a consensus. Studies were stratified based on whether they were outside or within the LT setting. In each group, studies with a comparative group of patients managed with standard of care are defined as managing liver disease without a specific AUD intervention. Among recipients of LT, separate analyses were performed for integrated and nonintegrated care models for the management of AUD. Data were extracted on study characteristics, population, intervention (behavioral or pharmacological), AUD care model (integrated or nonintegrated), and predefined outcomes (Table 1).

**TABLE 1:** Baseline characteristics of studies included in the analysis

References	Country	Study design population	Total N (intervention)	Intervention	AUD treatment model	Outcomes reported	No. of outcomes in treatment vs. control HR (95% CI)	Follow-up period
Patients with ALD outside LT setting (14 studies with N=92,276 with intervention in 33,177)								
Addolorato et al^20^	Italy	RCT AC	84 (42)	Baclofen	Integrated	Alcohol relapse	12 vs. 30 0.2 (0.1–0.9)^a^	12 wk
Ayyala et al^21^	USA	Retrospective ALD (AC 47%)	100 (63)	Naltrexone	Nonintegrated	Hospitalization	16 vs. 17 0.48 (0.24–0.95)	2 y
Bajaj et al^22^	USA	RCT AC	20 (10)	FMT	Nonintegrated	Alcohol relapse	1 vs. 7 0.09 (0.01–0.72)	6 mo
DeMartini et al^18^	USA	Pilot RCT AC	15 (8)	8 wk of text-message–based educational alcohol intervention	Integrated	Alcohol relapse	0 vs. 2 0.13 (0.01–2.71)	8 wk
Dhanda et al^23^	UK	RCT ALD (AC 54%)	54 (26) 20 (9)	Functional imagery training psychotherapy	Nonintegrated	Readmission Relapse	10 vs. 7 1.69 (0.64–4.46) 4 vs. 5 0.97 (0.26–3.68)	180 d
Higu^24^	Mexico	RCT Severe AH	79 (47) 135 (67)	Metadoxine	Nonintegrated	Alcohol relapse Patient mortality	12 vs. 13 0.57 (0.26–1.25) 24 vs. 50 0.33 (0.20–0.55)	6 mo
Morley et al^25^	Australia	RCT AUD (55% ALD)	93 (63)	Baclofen	Nonintegrated	Alcohol relapse	19 vs. 27 0.16 (0.08–0.30)	12 wk
Peeraphatdit et al^26^	USA	Retrospective multicenter Severe AH	294 (46)	Early alcohol rehabilitation within 30 d after hospital discharge for SAH	Nonintegrated	30-d readmission Alcohol relapse Patient mortality	7 vs. 83 0.41 (0.19–0.88) 3 vs. 95 0.14 (0.04–0.44) 4 vs. 91 0.20 (0.07–0.54)	2 y
Rabiee et al^27^	USA	Retrospective propensity-matched cohort AC	1732 (866)	Pharmacotherapy	Nonintegrated	Mortality	202 vs. 2330.80 (0.67–0.97)^a^	
Rogal et al^28^	USA	Retrospective multicenter AC	35,682 (5088) 32,693 (4411)	Behavioral therapy, either inpatient or outpatient	Nonintegrated	Patient mortality Hepatic decompensation	133 vs. 1203 0.66 (0.55–0.79) 287 vs. 3267 0.55 (0.49–0.62)	6 mo
Singal et al^29^	USA	Retrospective cohort AC or AH	53,319 (26,665)	Identifying AUD at discharge	Nonintegrated	30-d readmission	11,706 vs. 12,794 0.88 (0.86–0.91)	30 d
Vannier et al^11^	USA	Retrospective cohort AUD (12% ALD)	406 (105)	Pharmacological treatment	Nonintegrated	Hepatic decompensation	26 vs. 180 0.38 (0.25–0.57)^a^	
Vatsalya et al^30^	USA	RCT Moderate AH	46 (24)	LGG probiotic	Nonintegrated	Alcohol relapse	8 vs. 18 0.24 (0.10–0.57)	6 mo
Wang and Puglia^31^	Canada	Retrospective cohort AH or AC	297 (104)	SBIRT	Nonintegrated	90-d readmission	11 vs. 46 0.19 (0.07–0.50)	90 d
Patients with ALD within LT setting (11 studies with N=1623, AUD intervention in 657)								
Addolorato et al^14^	Italy	Retrospective single-center AC	92 (55)	Counseling sessions and pharmacotherapy delivered by alcohol addiction unit embedded within the LT team	Integrated	Alcohol relapse Patient mortality	9 vs. 13 0.41 (0.18–0.97) 8 vs. 14 0.33 (0.14–0.79)	5 y
Attillia et al^32^	Italy	Retrospective Single-center AC	87 (69)	Pretransplant screening of LT listed candidates listed for ALD and a monthly follow-up for 6 mo after LT	Integrated	Alcohol relapse	6 vs. 6 0.21 (0.06–0.68)^a^	5 y
Bjornsson et al^33^	Sweden	Retrospective Case–control Single-center AC	98 (58)	Structured management of AUD in the form of 12-step method delivered by an addiction specialist	Nonintegrated	Alcohol relapse	13 vs. 19 0.39 (0.19–0.80)	3 y
Carrique et al^34^	Canada	Prospective Early LT for ALD	155 (44)	Integrated multidisciplinary alcohol relapse prevention pilot program	Integrated	Alcohol relapse	3 vs. 18 0.40 (0.12–1.36)	1 y
Daniel et al^35^	France	Retrospective AC	611 (190)	Integrated multidisciplinary alcohol use monitoring	Integrated	Alcohol relapse Mortality	13 vs. 66 0.50 (0.27–0.90)^a^ 49 vs. 187 0.51 (0.37–0.70)	8 y
Erim et al^17^	Germany	Single center Prospective AC	100 (42)	Addiction group therapy in the form of 12 biweekly sessions over 6 mo before LT	Nonintegrated	Alcohol relapse	7 vs. 18 0.49 (0.20–1.18)	6 mo
Goswami et al^36^	USA	Prospective cohort AC	23 (18) 19 (14)	Telehealth AUD treatment program	Nonintegrated	Alcohol relapse (List) Alcohol relapse (LT)	2 vs. 3 0.13 (0.02–0.79) 4 vs. 4 0.21 (0.05–0.91)	12 wk
Magistri et al^19^	Italy	Retrospective Single-center AC	102 (28)	Involvement of a multidisciplinary team (clinical toxicologist, hepatologist, psychiatrist, and surgeon)	Nonintegrated	Alcohol relapse Patient mortality	5 vs. 17 0.75 (0.28–2.05) 0 vs. 22 0.05 (0.00–0.80)	5 y
Rodrigue et al^37^	USA	Retrospective 73% AC	115 (32)		Nonintegrated	Alcohol relapse	5 vs. 35 0.31 (0.12–0.79)	2 y
Weinrieb et al^38^	USA	Two-center randomized controlled trial AC on LT list	91 (46) 29 (13) 91 (46)	7 individual sessions of motivational enhancement therapy delivered by an addiction specialist over 6 mo	Nonintegrated	Alcohol relapse (List) Alcohol relapse (LT) Mortality	12 vs. 11 1.08 (0.47–2.45) 4 vs. 2 2.75 (0.50–15.10) 9 vs. 7 1.29 (0.48–3.46)	12 mo
Willenbring and Olson^39^	USA	Randomized clinical trial AC or AH	74 (38) 101 (48)	Integrated outpatient treatment in primary medical care, including techniques for addressing excessive drinking	Nonintegrated	Alcohol relapse Mortality	10 vs. 19 0.41 (0.19–0.88) 9 vs. 16 0.58 (0.25–1.31)	24 mo

^a^ Numbers reported in original papers.

Abbreviations: AH, alcohol-associated hepatitis; ALD, alcohol-associated liver disease; AUD, alcohol use disorder; LT, Liver transplantation; RCT, randomized controlled trial.

### Statistical analysis

Meta-analyses were used to compare AUD treatment to control in patients with ALD using random effects models. The meta-analysis estimates were obtained using the “meta” package in R program version 4.3.1.

When HRs were not directly reported in original publications, they were estimated by the ratio of the logarithm of event-free proportions between AUD treatment and control arms. For studies with zero events, 0.5 was added to prevent computational issues. In the 2 studies by Wang and Puglia, while the number of events was not fully reported, it could be calculated from the reported OR and information provided there, and further HR could be estimated.

The SE of the logarithm of the HR for each study was required to perform the meta-analysis. For studies where HRs and their 95% CIs were reported, the SE was determined using the reported HRs and their lower limits of the 95% CIs. For studies that did not report HRs, the variances were calculated based on the number of events and the number of nonevents in treatment and control arms.^40^

To evaluate the reliability and accuracy of this outlined estimation method,^40^ HRs and their corresponding 95% CIs were computed for 5 studies that provided HRs, and these calculated values were then compared to the originally reported HRs and 95% CIs. The comparison revealed that the estimated values closely approximated the reported ones (Supplemental Table S1, http://links.lww.com/HC9/B951).

Heterogeneity among different studies was determined by *I* ^2^ statistic. Pooled data with an *I* ^2^>50% was considered heterogeneous. The funnel plot was used to detect the potential publication bias. An asymmetrical funnel plot, particularly one showing an absence of small studies with nonsignificant effects, might suggest the presence of publication bias. Subgroup analyses were conducted to explore the sources of variability and examine the subgroup difference using the fixed-effects model. *p* values <0.05 were considered significant for all the analyses.

#### Protocol registration

The study protocol is registered at the Open Science Framework registry (https://doi.org/10.17605/OSF.IO/KYVJ2). We have provided a copy of the protocol in case reviewer/s are unable to access the OSF platform.

## RESULTS

### Studies characteristics

**FIGURE 1:**
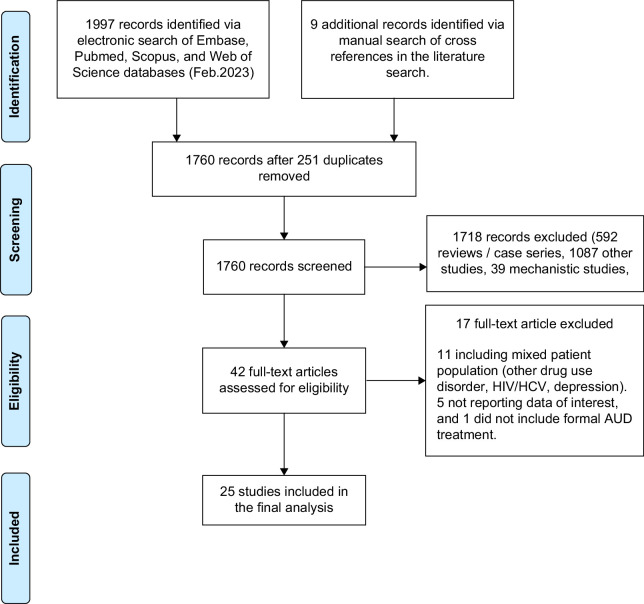
PRISMA figure on the selection of final studies for the analysis. Abbreviations: AUD, alcohol use disorder; PRISMA, Preferred Reporting Items for Systematic Reviews and Meta-Analyses.

Twenty-five eligible studies were analyzed (Figure 1), 14 on 92,276 (33,177 AUD treatment) patients with ALD outside the LT setting and 11 on 1623 (657 AUD treatment) within the LT setting (Table 1). Of 9 randomized studies, there was moderate bias in 6, low in 2, and high in 1 study (Supplemental Figures S1 and S2, http://links.lww.com/HC9/B950). Of 16 non-randomized studies, the bias risk was moderate in 10 and high in 5 studies (Supplemental Figures S3 and S4, http://links.lww.com/HC9/B950).

### Studies in patients with ALD outside the LT setting

A total of 92,276 patients with ALD (33,177 AUD treatment) were included in 14 studies (7 RCTs, 6 retrospective, and 1 prospective). AUD intervention was pharmacological in 8 (baclofen in 2 and 1 each on fecal matter transplant, naltrexone, LGG prebiotic, metadoxine, and variable drug in the remaining 2 studies) and behavioral in 5 (behavioral therapy in 1, SBIRT in 1, early alcohol rehabilitation within 30 days of discharge in 1, and telehealth/text-message in 2) studies. One study included both behavioral and pharmacological therapies^28^ (Table 1). Identifying AUD diagnosis at discharge was used as a surrogate of intervention in 1 study.^29^

#### Alcohol relapse

**FIGURE 2:**
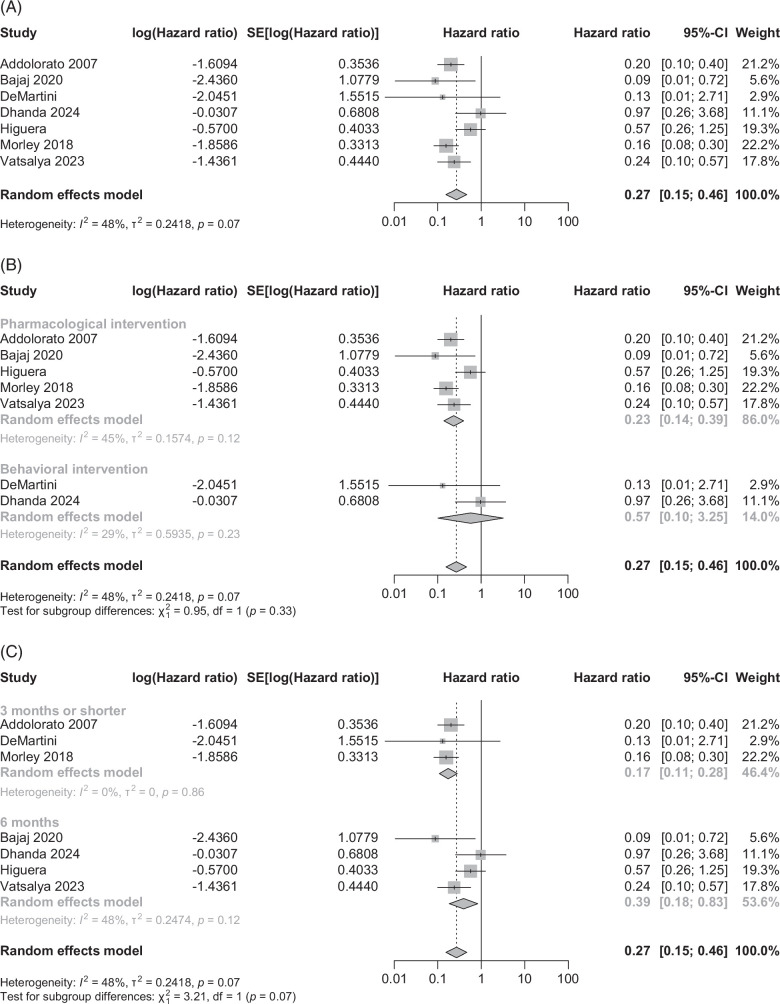
Alcohol relapse reported as HR (95% CI) on randomized controlled trials comparing (A) any treatment for alcohol use disorder versus no treatment, (B) subgroup analysis on medication versus behavioral intervention for alcohol use disorder, and (C) subgroup analysis on ≤3 versus 6 months of intervention for alcohol use disorder.

Seven RCTs on patients with ALD outside the LT setting examined the benefit of AUD treatment on relapse. The pooled effect size (95% CI) was 0.27 (0.15–0.46) in favor of treatment (Figure 2A), *p*<0.001. The pooled data showed moderate heterogeneity (*I* ^2^=48%, *p*=0.07) without publication bias (Supplemental Figure S5A, http://links.lww.com/HC9/B950).

#### Subgroup analyses

##### Pharmacological versus behavioral AUD treatment

Analysis of 5 RCTs after excluding 2 RCTs using behavioral intervention,^18^ the pooled HR was 0.23 (0.14–0.39) in favor of the intervention of 77% reduction in alcohol relapse (Figure 2B). The pooled data showed moderate heterogeneity (*I* ^2^=45%, *p*=0.12) without publication bias (Supplemental Figure S5B, http://links.lww.com/HC9/B950). However, the pooled effect size with 2 RCTs using behavioral intervention was not significantly different between intervention and control arms, pooled HR 0.57 (0.10–3.25). Interestingly, there was no difference between the subgroups on the pooled effect size (*χ* ^2^=0.95, *p*=0.33).

##### Treatment duration (≤3 vs. 6 months)

Analysis of 3 RCTs with ≤3 months of treatment, the pooled HR was 0.17 (0.11–0.28) in favor of the intervention (Figure 2C). The pooled data showed no heterogeneity (*I* ^2^=0%, *p*=0.86). The pooled effect size with 6 months of treatment in 4 RCTs was also in favor of intervention, HR 0.39 (0.18–0.83). However, there was moderate heterogeneity (*I* ^2^=48%, *p*=0.12). There was no significant difference between the 2 subgroups (*χ* ^2^=3.21, *p*=0.07).

#### Readmission

**FIGURE 3:**
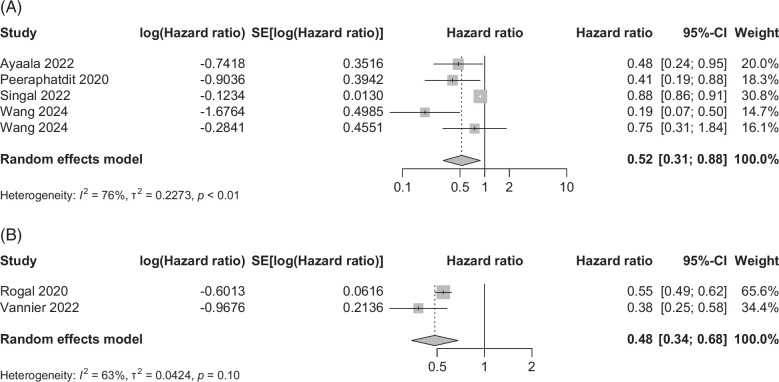
Pooled data of observational studies using the random effects model comparing alcohol use disorder treatment versus no treatment on (A) readmission and (B) hepatic decompensation.

A total of 5 observational (1 prospective) studies on patients with ALD outside the LT setting examined the benefit of AUD treatment on readmission. The pooled effect size (95% CI) was 0.52 (0.31–0.88) in favor of treatment (Figure 3A) with a 48% reduced risk of readmission, *p*=0.015. The pooled data showed moderate heterogeneity (*I* ^2^=76%, *p*<0.01) with publication bias (Supplemental Figure S6, http://links.lww.com/HC9/B950).

#### Hepatic decompensation

Two observational studies on patients with ALD outside the LT setting examined the benefit of AUD treatment on hepatic decompensation. The pooled effect size (95% CI) was 0.48 (0.34–0.68) (Figure 3B) tended to be in favor of treatment, *p*<0.001. The pooled data showed high heterogeneity (*I* ^2^=63%, *p*=0.10). Given retrospective design with several different medications for AUD (MAUD) could have accounted for heterogeneity. However, with only 2 studies in the analysis, we could not explore this further. However, with a large sample size with a protective effect size on hepatic decompensation in each study, we decided to retain the forest plot in this analysis. However, with only 2 studies, a funnel plot on publication bias is not reported.

#### Patient mortality

Three observational studies (1 prospective) on patients with ALD outside the LT setting examined the benefit of AUD treatment on patient mortality. The pooled effect size (95% CI) was 0.56 (0.29–1.07) (Supplemental Figure S7A, http://links.lww.com/HC9/B950), which was not significant, *p*=0.077. The pooled data showed high heterogeneity (*I* ^2^=77%, *p*=0.01) with publication bias (Supplemental Figure S7B, http://links.lww.com/HC9/B950).

### Studies among patients with ALD within an LT setting

A total of 1623 patients with ALD (657 received AUD treatment) were included in 11 studies (2 RCT, 2 prospective, and 7 retrospective). AUD intervention using an integrated multidisciplinary model with addiction and hepatology teams in the same clinic or program was used to monitor alcohol use and provide AUD intervention in 4 studies (Table 1).

### Analysis of patients with ALD listed for and awaiting LT

#### Alcohol relapse

Two RCTs on patients with ALD awaiting LT examined the benefit of AUD treatment on alcohol relapse. The pooled effect size (95% CI) was not significant, 0.66 (0.25–1.70, *p*=0.387). The pooled data showed high heterogeneity (*I* ^2^=65%, *p*=0.09). Two observational studies on patients with ALD awaiting LT examined the benefit of AUD treatment on alcohol relapse. The pooled effect size (95% CI) was not significant, 0.32 (0.09–1.09, *p*=0.068). The pooled data showed moderate heterogeneity (*I* ^2^=41%, *p*=0.19). Given only 2 studies in these analyses, forest plots on pooled effect size and funnel plots for publication bias are not presented.

#### Patient survival

Two RCTs on patients with ALD awaiting LT examined the benefit of AUD treatment on patient mortality. The pooled effect size (95% CI) was not significant, 0.82 (0.38–1.79, *p*=0.619) (Supplemental Figure S8, http://links.lww.com/HC9/B950). The pooled data showed no heterogeneity (*I* ^2^=33%, *p*=0.220). With only 2 studies, a funnel plot on publication bias is not reported. None of the observational studies among patients listed for ALD examined the effect of AUD treatment on patient mortality.

### Analysis of LT recipients with ALD

#### Alcohol relapse

**FIGURE 4:**
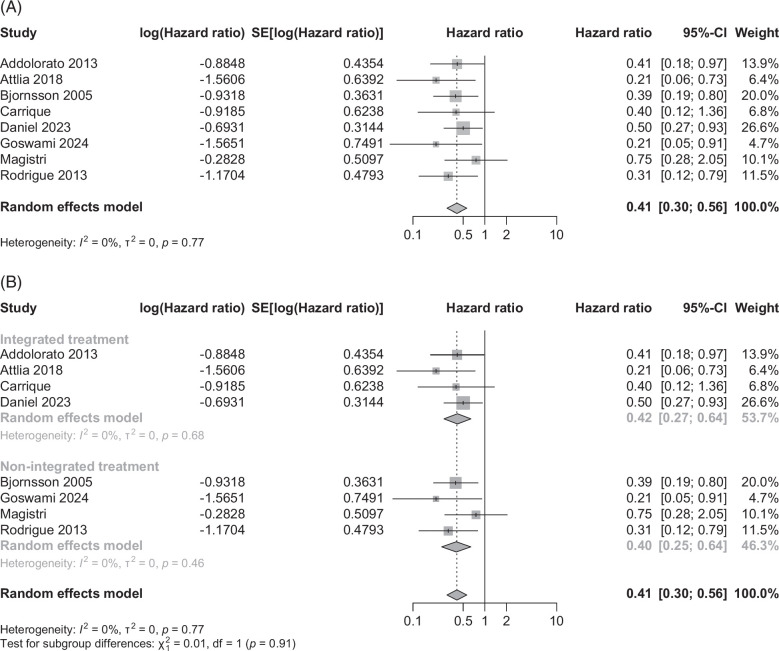
Pooled data reported as HR (95% CI) on alcohol relapse for (A) observational studies using behavioral intervention versus no treatment in liver transplant recipients with alcohol-associated liver disease on alcohol relapse and (B) subgroup analysis comparing integrated versus nonintegrated model for the treatment of alcohol use disorder.

Eight observational studies among LT recipients with ALD examined the benefit of AUD treatment on alcohol relapse. The pooled effect size (95% CI) showed a 59% reduction in alcohol relapse with AUD treatment, 0.41 (0.30–0.56, *p*<0.001) (Figure 4A). The pooled data showed no heterogeneity (*I* ^2^=0%, *p*=0.77) without publication bias (Supplemental Figure S9, http://links.lww.com/HC9/B950).

### Subgroup analyses

#### Integrated versus nonintegrated AUD treatment

Analysis of 4 observational studies using an integrated multidisciplinary care model of AUD treatment (addiction team embedded within the LT center) showed the benefit of AUD treatment. The pooled data showed a 58% reduction in alcohol use with AUD treatment, 0.42 (0.27–0.64, *p*<0.001) (Figure 4B). The pooled data showed no heterogeneity (*I* ^2^=0%, *p*=0.68) without publication bias (Supplemental Figure S10A, http://links.lww.com/HC9/B950). Analysis of the remaining four observational studies using a nonintegrated multidisciplinary care model of AUD treatment also showed the benefit of AUD treatment. The pooled data showed a 60% reduction in alcohol use with AUD treatment, 0.40 (0.25–0.64, *p*<0.001) (Figure 4B). The pooled data showed no heterogeneity (*I* ^2^=0%, *p*=0.46) without publication bias (Supplemental Figure S10B, http://links.lww.com/HC9/B950). There was no subgroup difference indicated between the 2 subgroups (*χ* ^2^=0.01, *p*=0.91).

#### Study region (Europe vs. USA)

Analysis of 5 studies from Europe showed the pooled HR of 0.45 (0.31–0.64) in favor of intervention (Supplemental Figure S11A, http://links.lww.com/HC9/B950). The pooled data showed no heterogeneity (*I* ^2^=0%, *p*=0.60). The pooled effect size on 3 studies from the United States was also in favor of intervention, HR 0.31 (0.16–0.60), without heterogeneity (*I* ^2^=0%, *p*=0.80). There was no significant difference between the subgroups (*χ* ^2^=0.91, *p*=0.34).

### Patient survival

**FIGURE 5:**
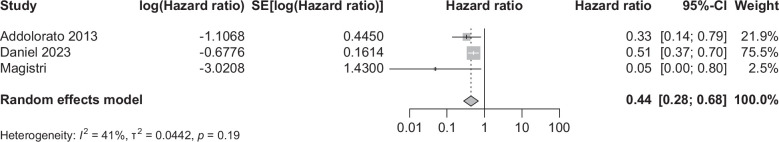
Pooled data reported as HR (95% CI) on patient mortality in observational studies using behavioral intervention versus no treatment in liver transplant recipients for alcohol-associated liver disease.

Three observational studies on LT recipients with ALD examined the benefit of AUD treatment on patient mortality. The pooled effect size (95% CI) tended to be in favor of AUD treatment, 0.44 (0.28–0.68, *p*<0.001) (Figure 5). The pooled data showed moderate heterogeneity (*I* ^2^=41%, *p*=0.19) with publication bias (Supplemental Figure S11B, http://links.lww.com/HC9/B950).

## DISCUSSION

The main findings are that treatment of AUD in patients with ALD outside the LT setting reduces alcohol relapse and liver-related outcomes. Among recipients of LT, the observational data shows reduced alcohol relapse and patient mortality with AUD treatment, both with and without a multidisciplinary integrated approach.

In a meta-analysis of 39 randomized trials on 15,974 adults, an AUD intervention was associated with 33% reduction in heavy drinking, and 40% reduction in drinking outside recommended limits at 6–12 months follow-up.^41^ However, this analysis was in individuals with AUD without obvious liver disease. In this meta-analysis, we included patients with pure ALD, unlike a previous report from our group, which also included patients with ALD and concomitant HCV infection.^42^ Further, the current meta-analysis included 5 studies with pharmacological treatment of AUD, unlike 4 in another meta-analysis on MAUD in patients with ALD.^43^

An important observation from this pooled data is the rarity of the use of MAUD. Despite 581,556 cases of cirrhosis (135,879 decompensated) in the United States in 2017, only 346 patients with ALD patients had been enrolled in randomized clinical trials until this meta-analysis, with 180 receiving an active medication for AUD. Further, these RCTs only examined the effect of the intervention on alcohol relapse and not on liver outcomes. Moreover, there were no RCTs investigating AUD treatment in LT recipients with ALD. In the real world also, the use of AUD treatment is utilized rarely in patients with ALD, with only 9%–30% of these patients receiving AUD treatment (behavioral, pharmacological, or both).^44^ Rarity of simultaneous management of AUD in patients with ALD is due to several barriers at the level of patients, clinicians, and administrative implementation, which explains inconclusive data in our analysis on the benefit of AUD intervention in patients with liver disease.

Despite limited data, an AUD intervention was associated with reduced readmission and liver decompensation. Although the effect size was in favor of intervention for patient mortality, the pooled data from 3 observational studies was not significant for this outcome.

Our analysis of observational data among LT recipients with ALD showed the benefit of AUD treatment in reducing alcohol relapse and patient mortality. These benefits were observed with both integrated multidisciplinary and nonintegrated approaches to AUD treatment. In a previous systematic review of 13 studies, integrated management of AUD (cognitive behavioral or motivational enhancement therapy) was reported to be better at achieving abstinence.^45^

Although we were unable to analyze the patient mortality comparing heavy users versus abstinent recipients of LT, several studies earlier have shown a worse patient survival at 5–10 years follow-up among recipients of LT who relapse to heavy alcohol use compared to abstinent or those with occasional slips.^6^^,^^46^^,^^47^

There are several strengths of our meta-analysis like most recent literature, sound methodology by statistical experts, and analysis of RCTs and observational data separately. However, we do acknowledge the limitations of our study. For example, analysis on patients with liver disease was limited due to several of the studies being observational with a high level of bias, especially studies in recipients of LT, variability in the duration of treatment, variations on the type of AUD treatment, and on monitoring of alcohol use, and lack of a comparator group, especially on the outcome of decreased alcohol use. There is also a scarcity of studies, especially on the assessment of MAUD in patients with ALD, which limits evidence-based guidance to providers in clinical practice on the use of medications to treat AUD in patients with ALD. This becomes even more relevant given several barriers to the use of MAUD in ALD patients, especially hesitancy of gastroenterologists and hepatologists on the use of MAUD considering safety concerns and belief that they are not adequately trained on the use of these medications in ALD patients.^12^^,^^48^ It should also be noted that even this scarce data comes from developed high-income countries and the Western world, limiting the generalizability of these findings to developing countries with low to middle-income levels. Further, the exclusion of publications reported only as abstracts or in a language other than English may have impacted publication bias. Although, we were able to perform subgroup analyses based on the type of AUD intervention (concomitant or integrated care model), the small sample size limited evaluation of other variables such as type of AUD intervention, definitions of alcohol relapse (occasional slip vs. moderate use vs. heavy use), and monitoring tools for alcohol use (self-report or supplemented with different alcohol use biomarkers).

However, we feel that despite these limitations, our meta-analysis provides a set of important observations on AUD treatment in patients with ALD. First, we observed a scarcity of data on AUD treatment in ALD patients outside and within the LT setting, especially on medications. Second, outside the LT setting, only database studies have examined liver-related outcomes such as readmission and hepatic decompensation. Third, the pooled data, although heterogeneous and inclusive of the varied study population, the pooled data is homogeneous, with 5.3% mortality in intervention and 10.7% in the untreated group. These numbers are relevant in sample size calculation and in designing future clinical trials.

## CONCLUSIONS

Available data on AUD treatment in patients with ALD improves abstinence and liver-related outcomes both outside and within LT settings. However, the data on liver outcomes of decompensation and patient mortality should be interpreted with caution given the small number of studies with moderate quality and small sample size. RCTs are needed to examine (a) medications in ALD patients to examine the benefit of alcohol relapse and patient outcomes and (b) the benefit of integrated multidisciplinary treatment to manage the dual pathology (AUD and liver disease).

RCTs are also needed with integrated design of medications targeting liver disease and AUD, with the goal of improving long-term patient outcomes, and examine association with benefit on AUD with liver outcomes.^49^ These studies should target advanced forms of cirrhosis and/or AH first to establish guidance for providers and bridge the knowledge gap of alcohol use threshold associated with negative outcomes such as maintenance of complete abstinence, although a desired goal is difficult to achieve in patients with AUD, including those with ALD.^8^^,^^50^

## Supplementary Material
