# Supplementary material for: Treatment of alcohol use disorder in alcohol-associated liver disease: A meta-analysis
Source: Hepatol Commun. 2025 Apr 30;9(5):e0686. doi: 10.1097/HC9.0000000000000686 (PMC12045530; doi:10.1097/HC9.0000000000000686)
Supplement: Supplementary file 1 [file hc9-9-e0686-s001.docx]

**Literature search strategy**

The following keywords were used in the database search: “*Ethanol, Alcohol use, Alcohol use disorder, Alcohol dependence, Alcohol-associated, Alcoholism, Alcohol-related disorders, Alcohol-related liver disease, Alcohol-induced disorders, Alcohol withdrawal syndrome, AWS, Substance-related disorders, Liver diseases, Fatty liver, Liver transplantation, Cirrhosis, Liver cirrhosis, Multidisciplinary team, Integrative management, Addiction, Addiction team, Behavior therapy, Drug therapy, Psychotherapy, Motivational therapy and Pharmacological therapy*”. In addition, authors performed a manual search of references from the bibliography in the retrieved articles. The strategy used by our librarian to combine search words was as follows: *(((Psychotherapy[Mesh] OR behavior therapy[Mesh]] OR biofeedback, psychology[Mesh] OR cognitive therapy[Mesh] OR mindfulness[Mesh] OR relaxation therapy[Mesh] OR meditation [Mesh] OR feedback, psychological [Mesh] OR “combined modality therapy”[Mesh] OR counseling[Mesh] OR motivational interviewing[Mesh] OR “motivation therapy[tiab]” OR “motivational intervention”[tiab] OR pharmacotherapy[tiab] OR “behavior therapy”[tiab] OR “behavior modification”[tiab] OR pharmacotherapy[tiab] OR psychotherapy[tiab] OR behavioral[tiab] OR psychiatric rehabilitation[Mesh] OR “integrated care”[tiab] OR “behavioral intervention”[tiab] OR “integrated”[tiab] OR “integrated care”[tiab] OR ”intervention”[tiab])) AND (Liver diseases[Mesh] OR “liver diseases/therapy”[Mesh] OR liver cirrhosis[Mesh] OR liver diseases, alcoholic[Mesh] OR liver cirrhosis, alcoholic[Mesh] OR liver[Mesh] OR liver function tests[Mesh] OR “liver disease*[tiab] OR ALD[tiab] OR “alcoholic liver disease”[tiab] OR “alcohol associated liver disease[tiab] OR “liver transplantation”[Mesh] OR “hepatitis c, chronic”[Mesh] OR “liver cirrhosis, alcoholic”[Mesh] OR “alcoholic hepatitis”[tiab] OR HCV[tiab])) AND (Alcohol drinking[Majr] OR binge drinking[Majr] OR alcoholic intoxication[Majr] OR alcoholism[Majr] OR "alcohol abstinence”[Majr] OR “alcohol related”[tiab] OR “alcohol-related”[tiab] OR “alcohol associated”[tiab] OR “alcohol-associated”[tiab] OR “alcohol use disorder”[tiab] OR AUD[tiab] OR “alcohol abuse”[tiab] OR “alcohol dependence”[tiab] OR “alcohol withdrawal syndrome”[tiab] OR AWS[tiab] OR “alcohol abstinence”[tiab] OR “alcohol use”[tiab] OR “substance abuse, intravenous”[Majr] OR “substance abuse”[tiab]) Sort by: Best Match Filters: Humans; English.*


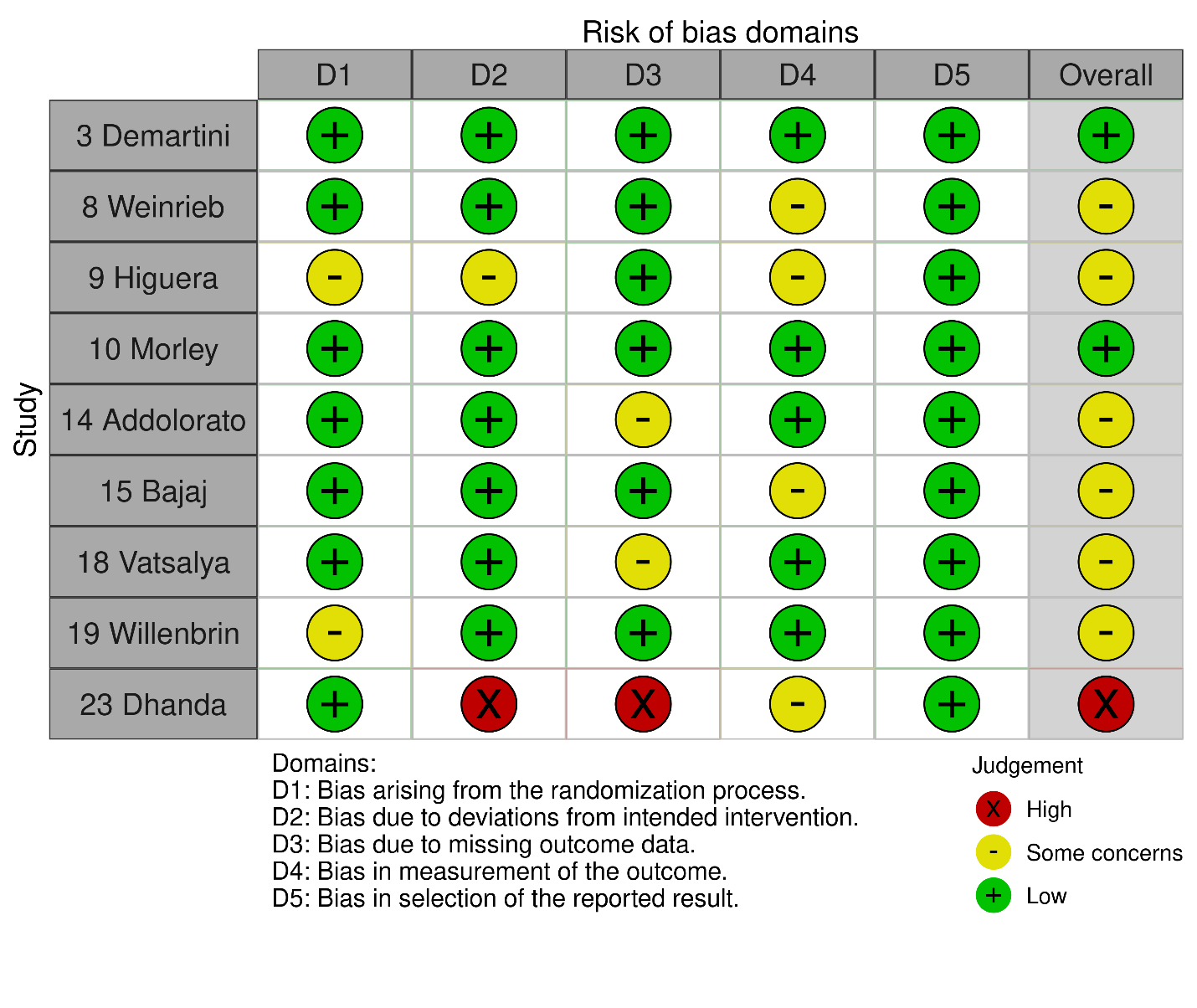


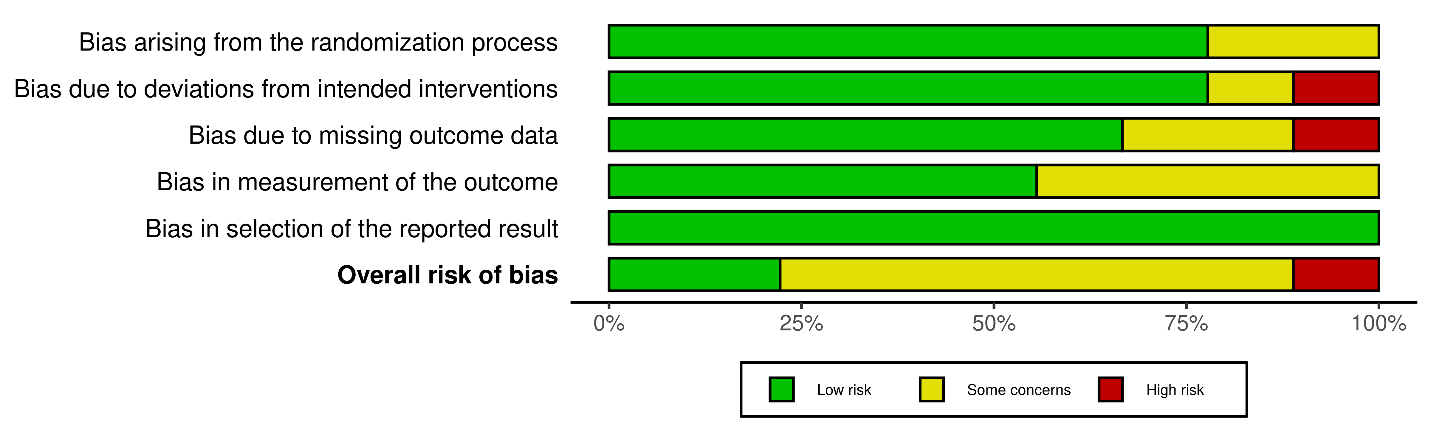


**Supplementary Figures 1 and 2**: Quality assessment of randomized studies.


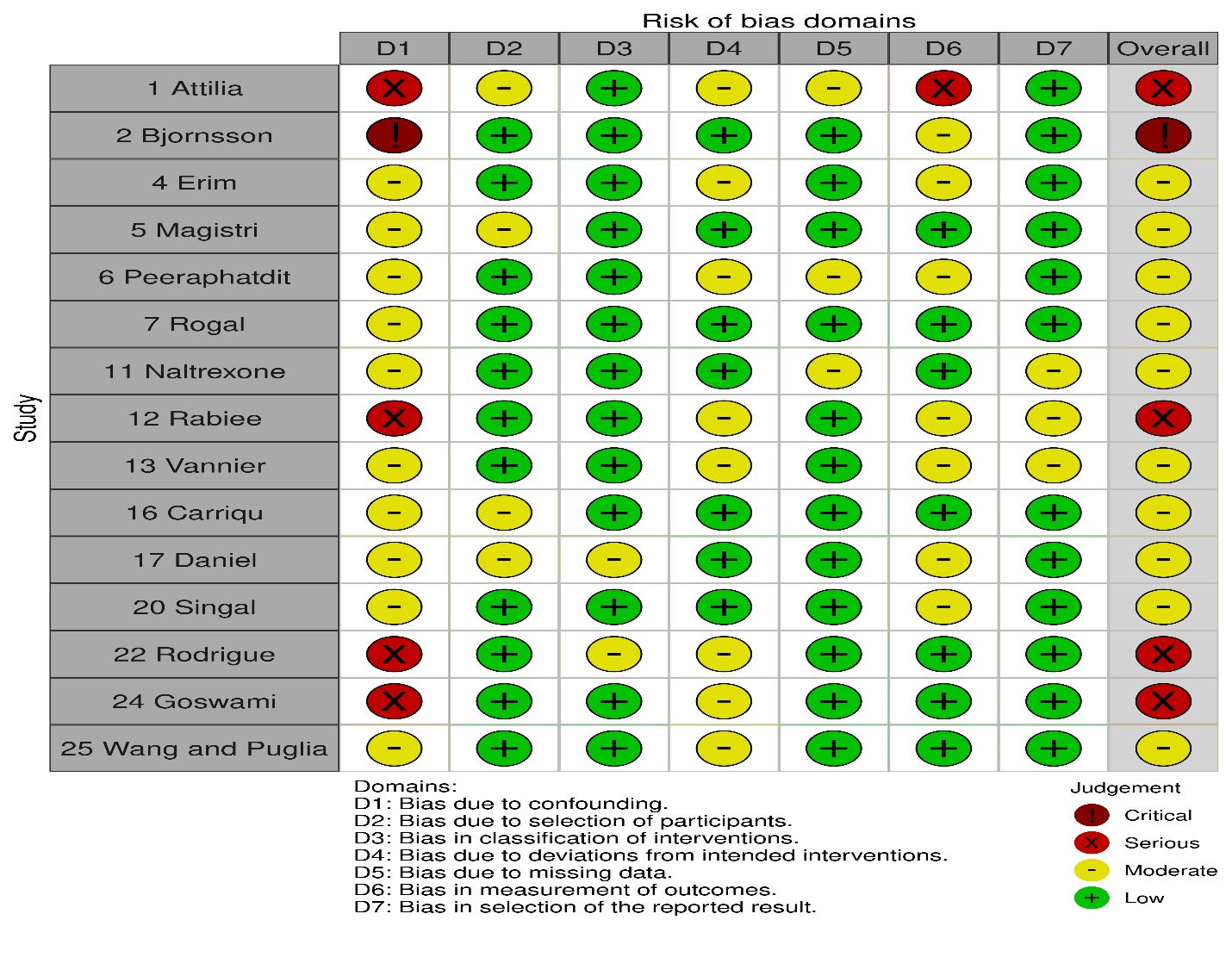


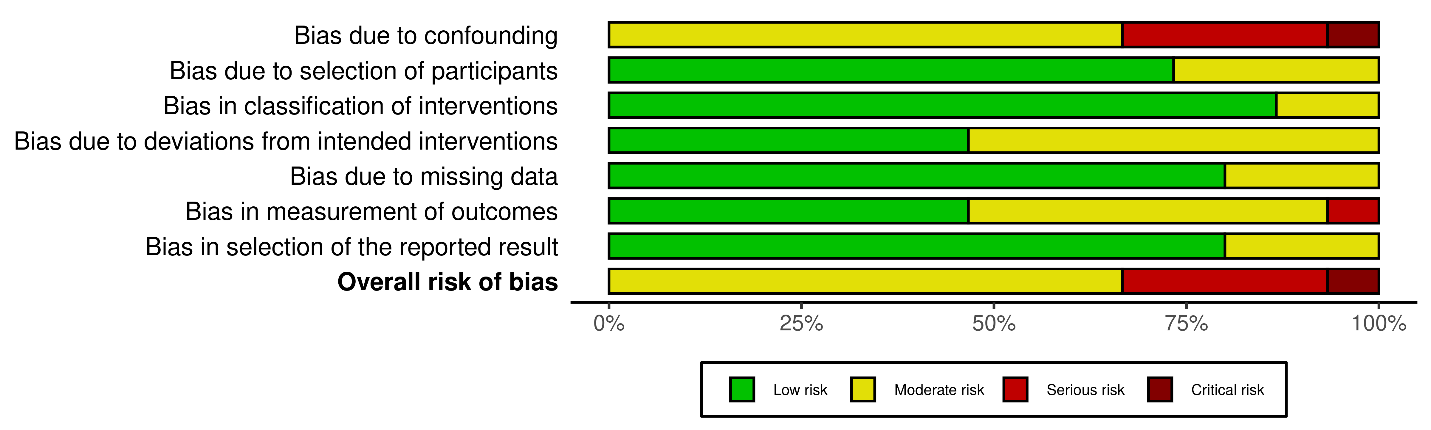


**Supplementary Figures 3 and 4**: Quality assessment of non-randomized studies.


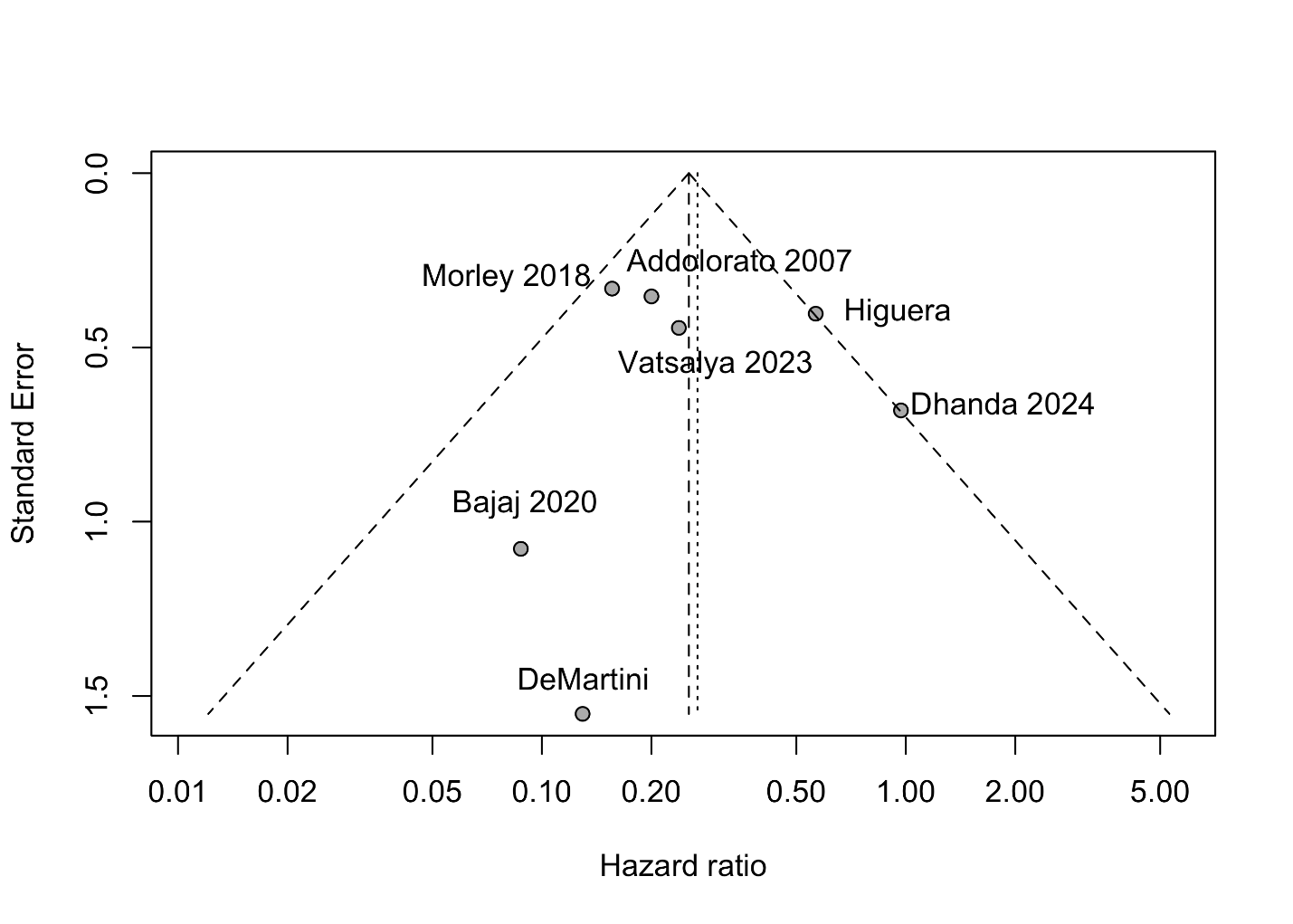


**Supplementary Figure 5A** Funnel plot for alcohol relapse based on randomized controlled trials comparing any treatment for alcohol use disorder vs. no treatment


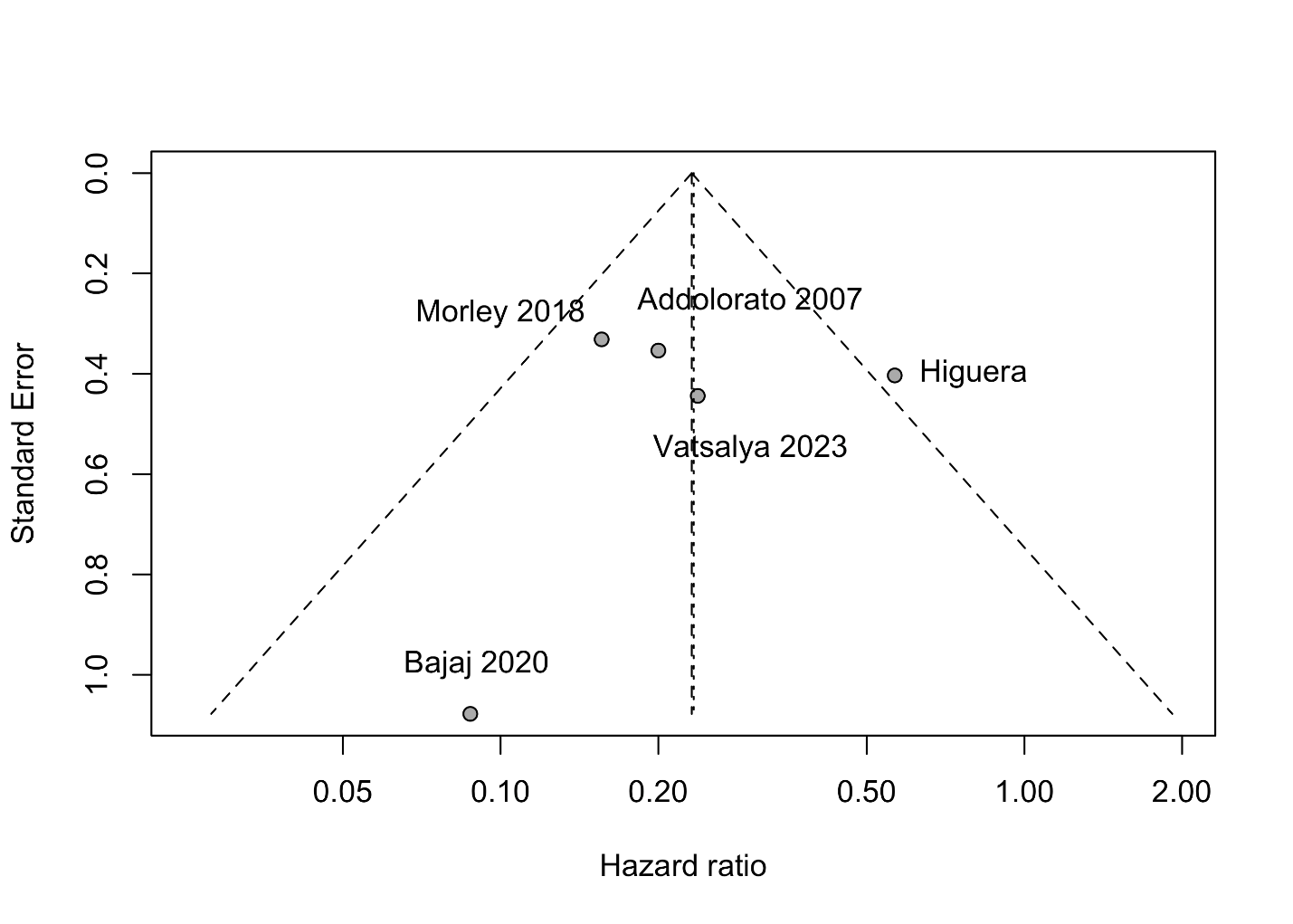


**Supplementary Figure 5B** Funnel plot for alcohol relapse based on randomized controlled trials comparing medication for alcohol use disorder vs. no medication.


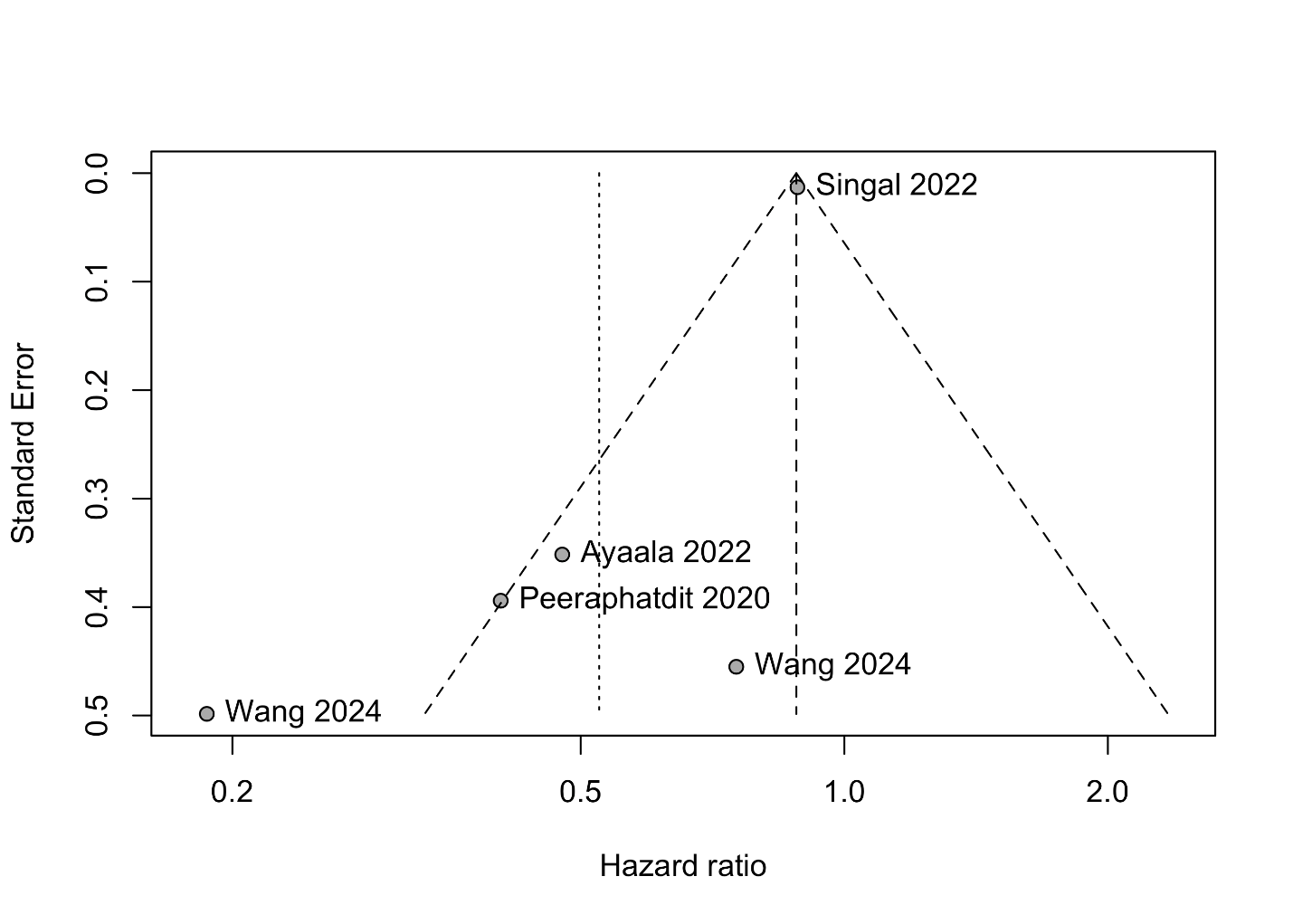


**Supplementary Figure 6** Funnel plot for observational studies comparing alcohol use disorder treatment vs. no treatment on readmission.

**
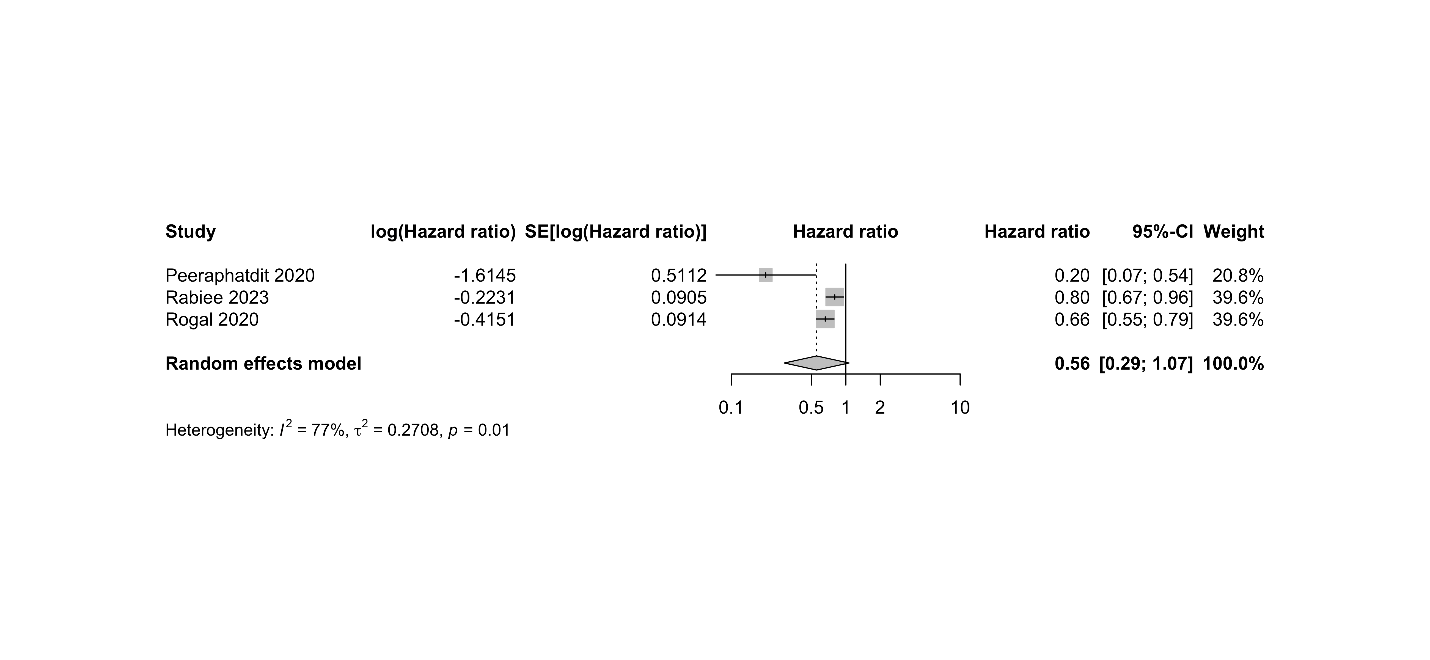
**

**Supplementary Figure 7A**: Pooled data of observational studies using the random effects model comparing alcohol use disorder treatment vs. no treatment on patient mortality.


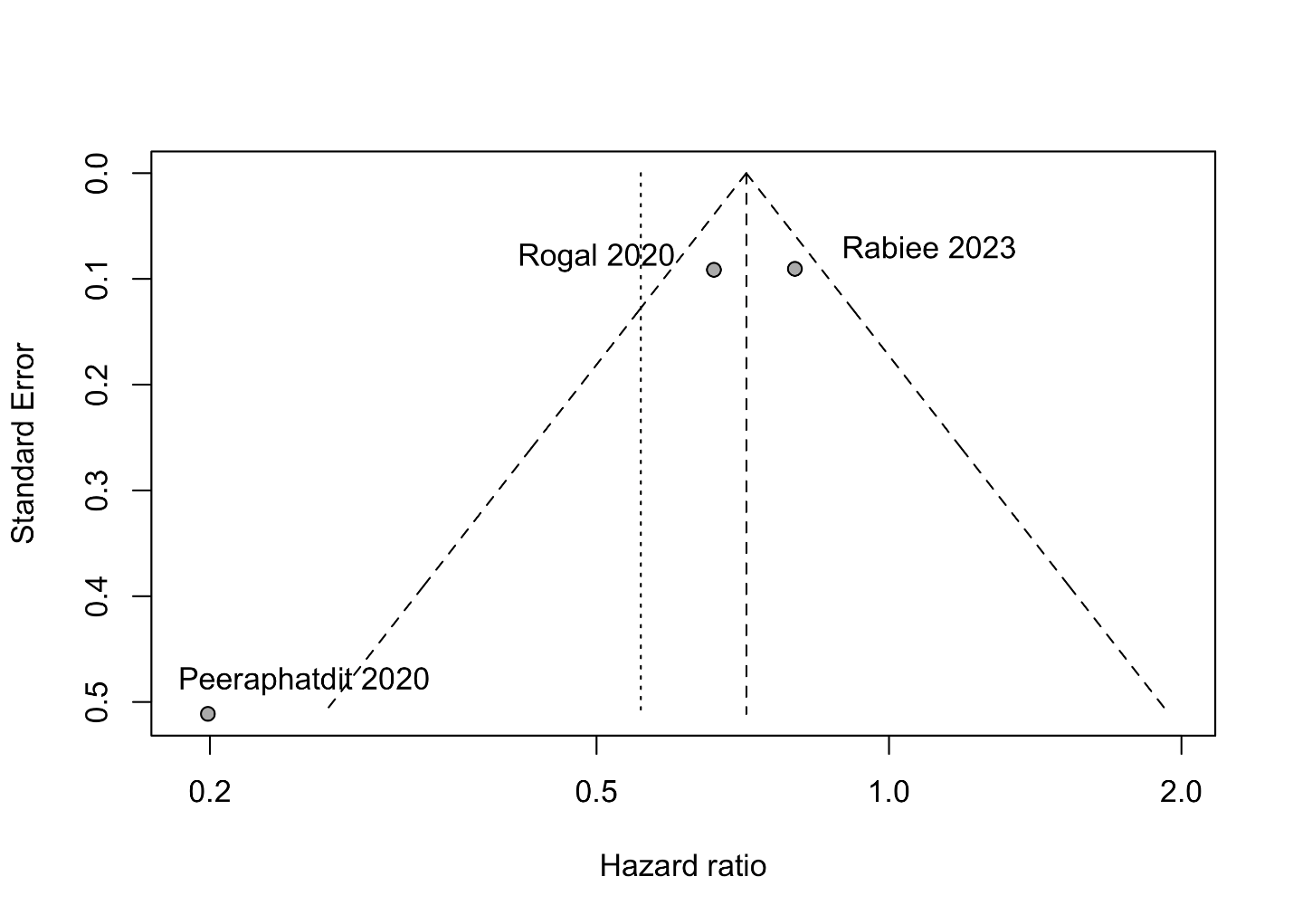


**Supplementary Figure 7B** Funnel plot for pooled data of observational studies using the random effects model comparing alcohol use disorder treatment vs. no treatment on patient mortality.


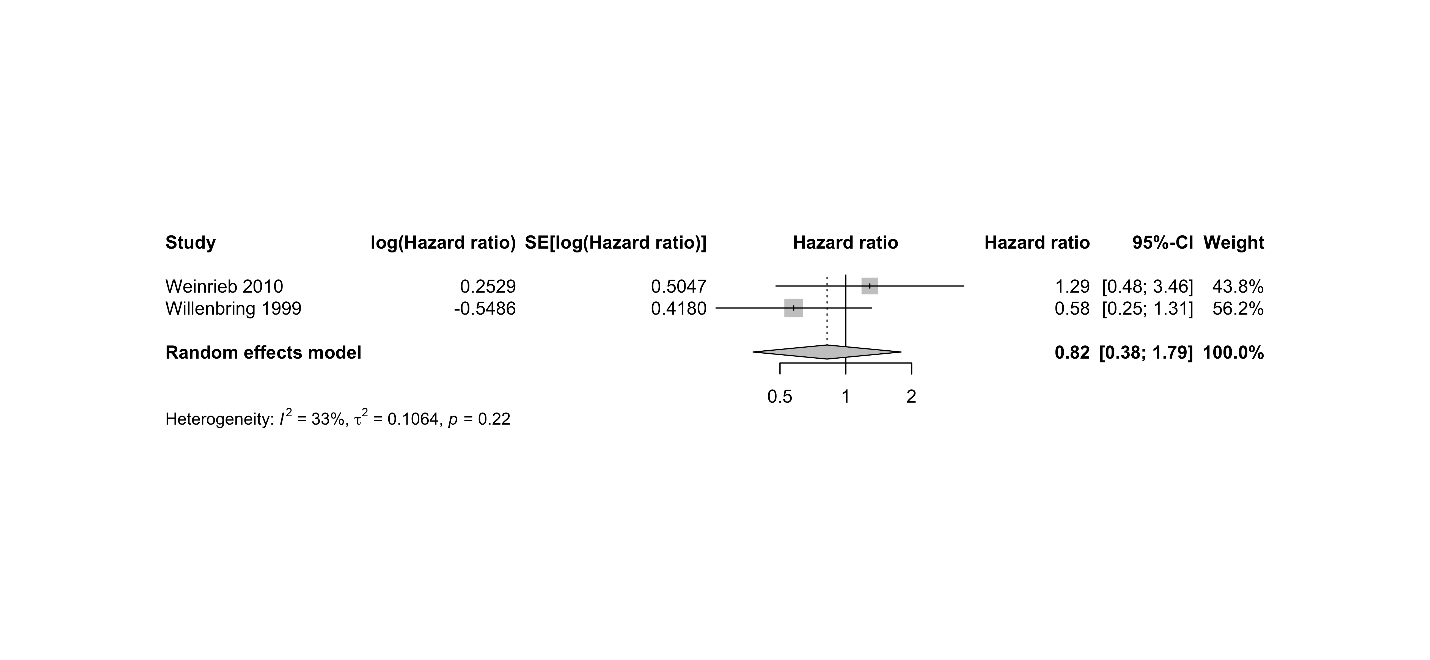


**Supplementary Figure 8**: Pooled data of randomized controlled studies on alcohol relapse using the random effects model comparing alcohol use disorder treatment vs. no treatment in patients with alcohol-associated liver disease awaiting liver transplant.


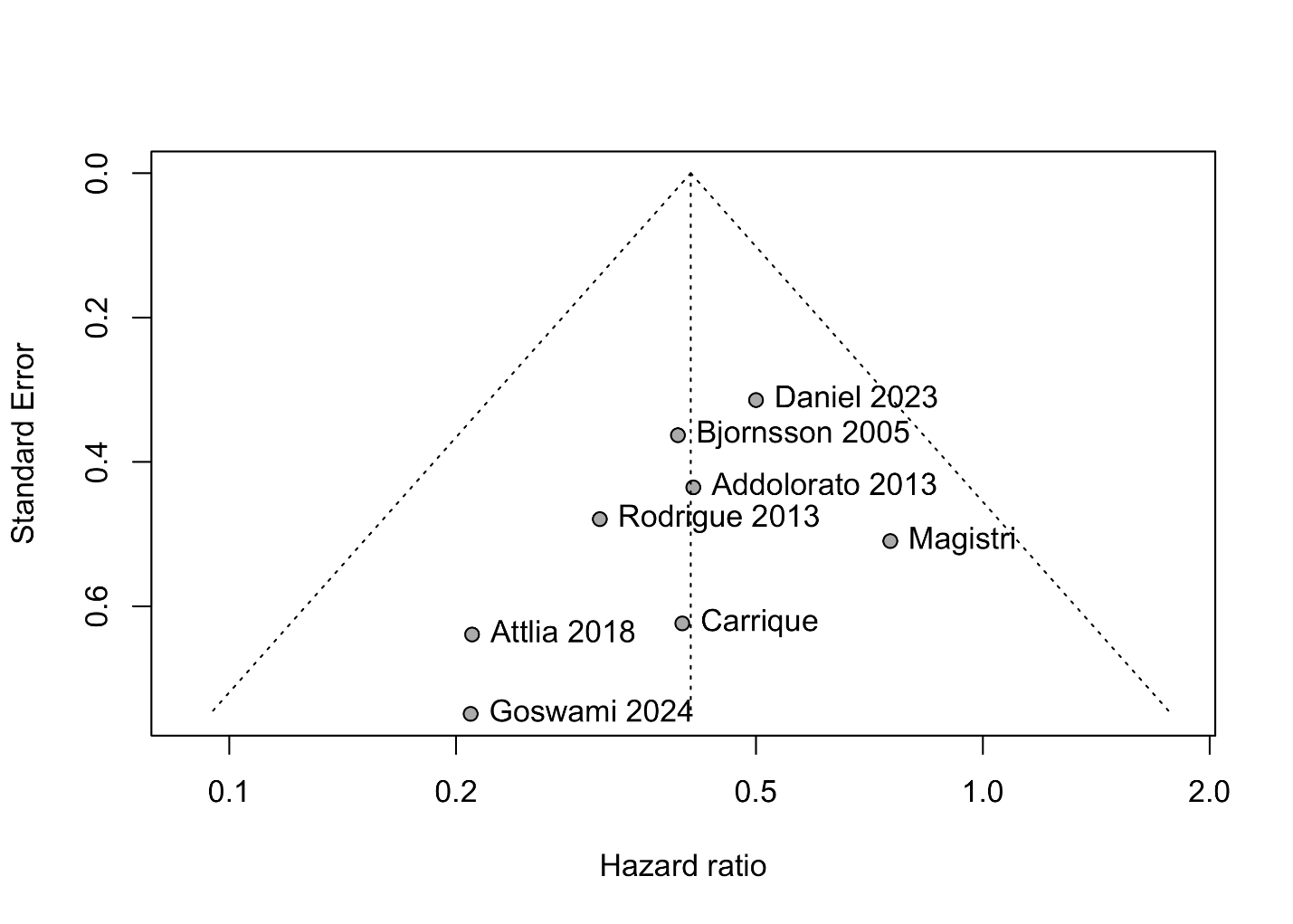


**Supplementary Figure 9** Funnel plot for alcohol relapse based on observational studies in liver transplant recipients for alcohol-associated liver disease comparing non-pharmacological treatment for alcohol use disorder vs. no treatment.


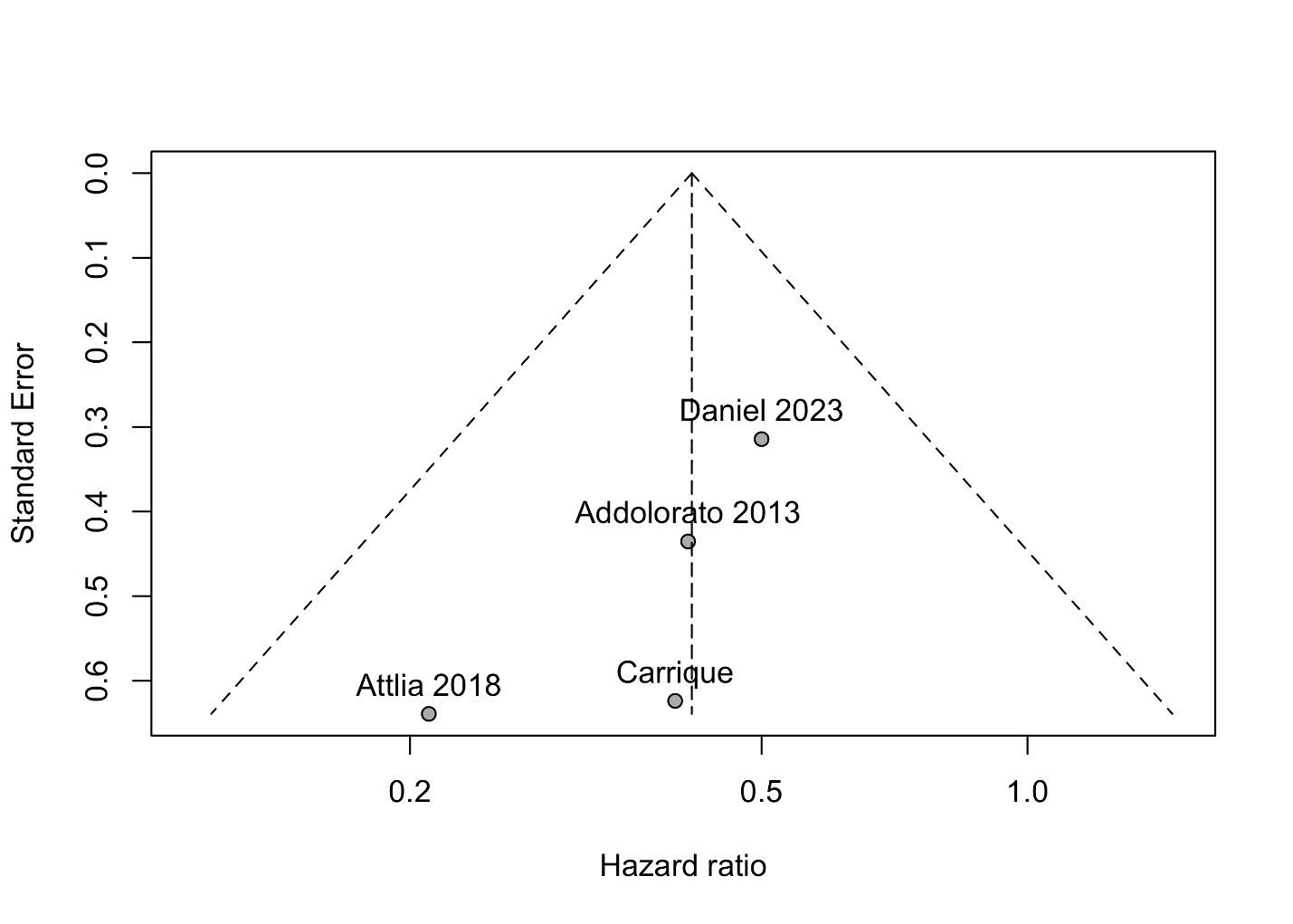


**Supplementary Figure 10A** Funnel plot for alcohol relapse on observational studies in liver transplant recipients for alcohol-associated liver disease comparing non-pharmacological treatment for alcohol use disorder vs. no treatment using integrated multidisciplinary care model.


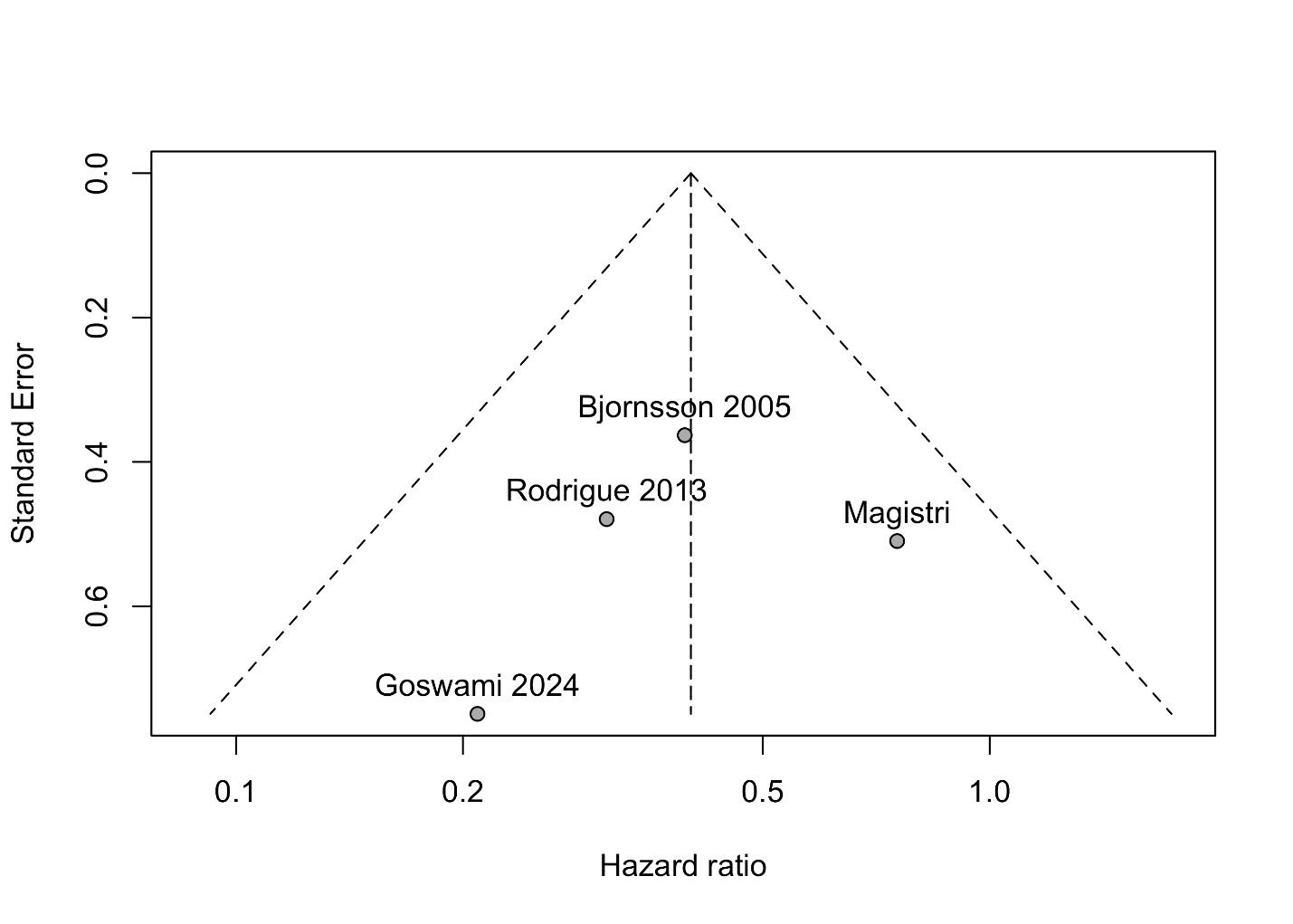


**Supplementary Figure 10B** Funnel plot for alcohol relapse on observational studies in liver transplant recipients for alcohol-associated liver disease comparing non-pharmacological treatment for alcohol use disorder vs. no treatment using non-integrated care model.


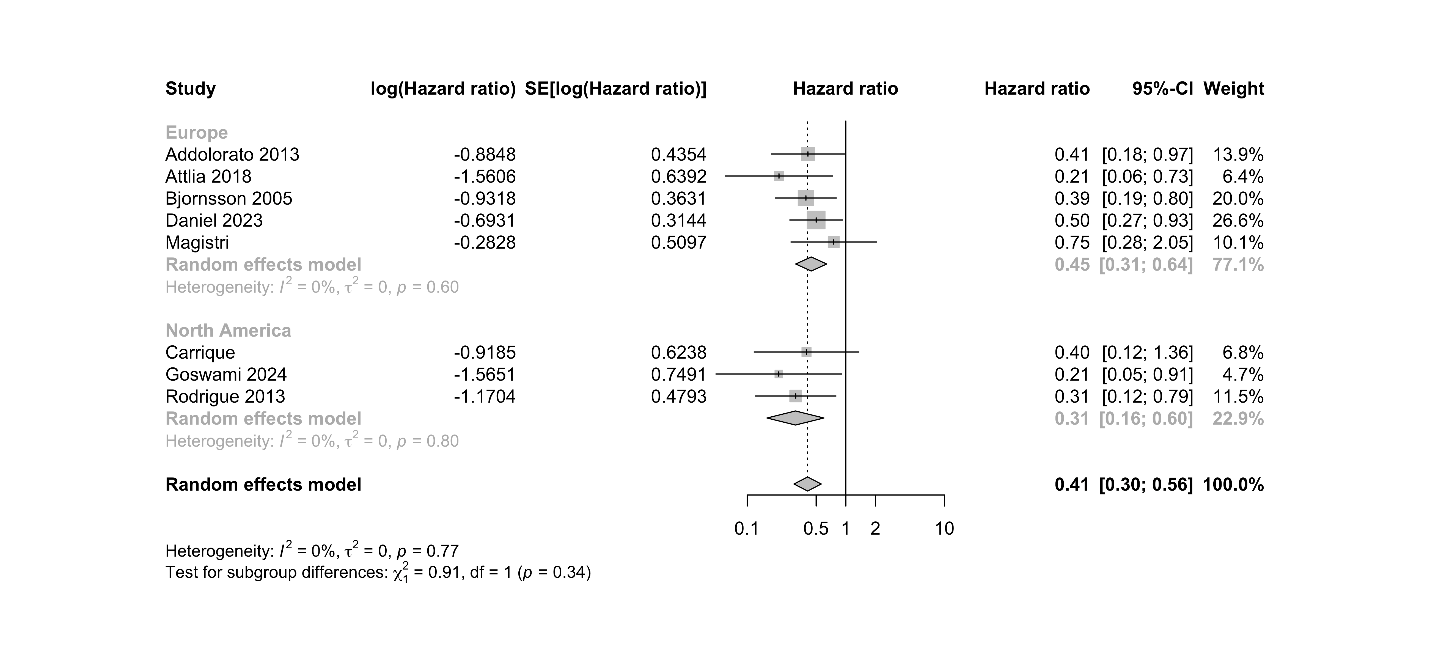


**Supplementary Figure 11A** Forest plot for alcohol relapse on observational studies in liver transplant recipients: subgroup analysis comparing studies from Europe and studies from the USA.


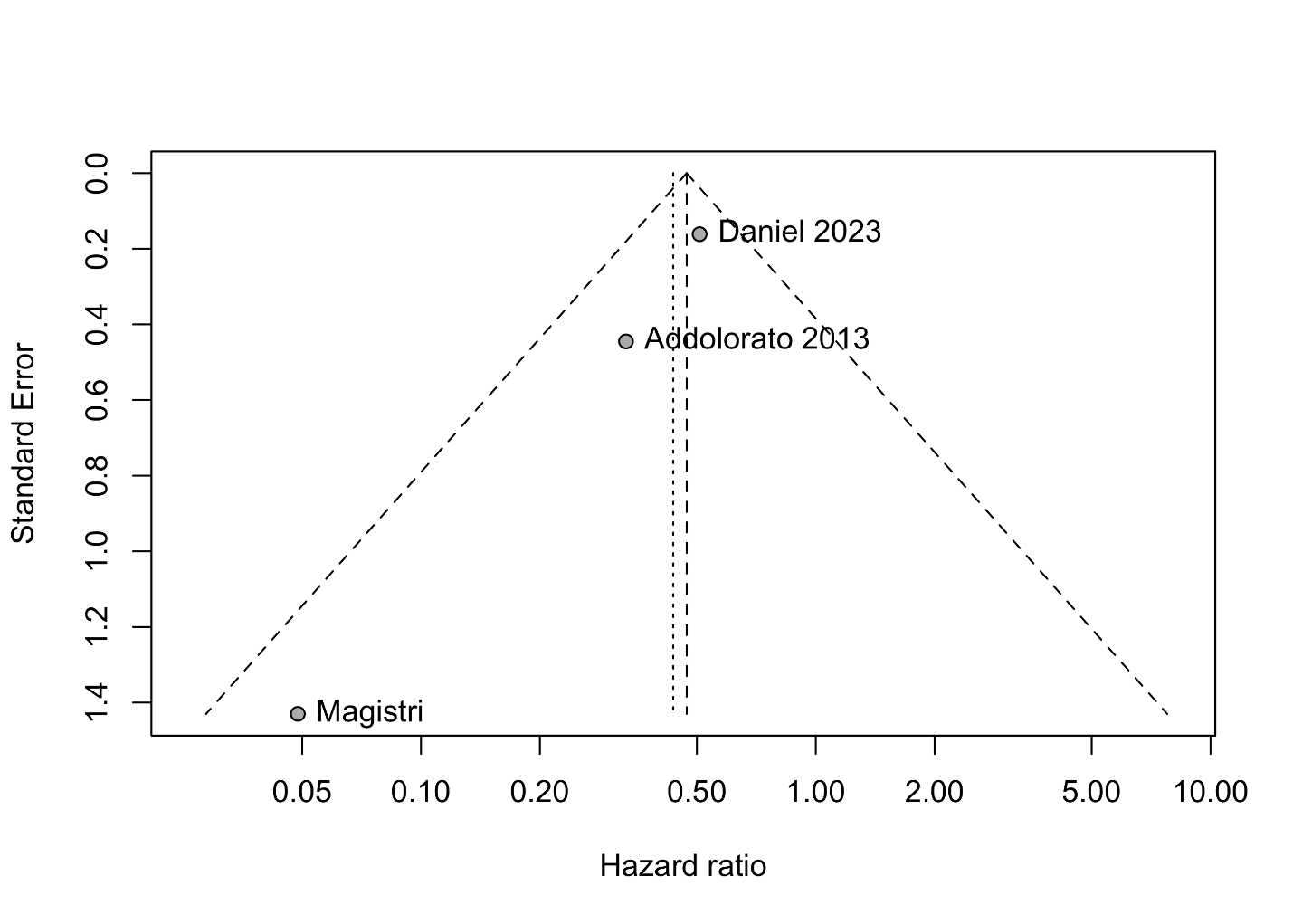


**Supplementary Figure 11B** Funnel plot for patient mortality on observational studies in liver transplant recipients for alcohol-associated liver disease comparing non-pharmacological treatment for alcohol use disorder vs. no treatment.
